# Correction: Functional human 3D microvascular networks on a chip to study the procoagulant effects of ambient fine particulate matter

**DOI:** 10.1039/c8ra90041h

**Published:** 2018-05-18

**Authors:** Yan Li, Qing-Meng Pi, Peng-Cheng Wang, Lie-Ju Liu, Zheng-Gang Han, Yang Shao, Ying Zhai, Zheng-Yu Zuo, Zhi-Yong Gong, Michael Mak, Xu Yang, Yang Wu

**Affiliations:** Key Laboratory for Deep Processing of Major Grain and Oil (Wuhan Polytechnic University), Ministry of Education, College of Food Science and Engineering, Wuhan Polytechnic University Wuhan 430023 PR China wuyangwhpu@163.com +86-27-83956793 ext. 208 +86-13871548015; Hubei Key Laboratory of Genetic Regulation and Integrative Biology, College of Life Sciences, Central China Normal University Wuhan 430079 PR China yanxgu@mail.ccnu.edu.cn +86-27-67861936 +86-13871361954; Harvard-MIT Division of Health Sciences and Technology, Massachusetts Institute of Technology Cambridge MA 02139 USA; Department of Biomedical Engineering, School of Engineering & Applied Science, Yale University New Haven 06520 USA; Department of Orthopedic Surgery, Shanghai Jiaotong University Affiliated Sixth People’s Hospital, Shanghai Jiaotong University Shanghai 200233 PR China; School of Biology and Pharmaceutical Engineering, Wuhan Polytechnic University Wuhan 430023 PR China

## Abstract

Correction for ‘Functional human 3D microvascular networks on a chip to study the procoagulant effects of ambient fine particulate matter’ by Yan Li *et al.*, *RSC Adv.*, 2017, **7**, 56108–56116.

The authors regret the omission of one of the authors, Michael Mak, from the original manuscript. The corrected list of authors and affiliations for this paper is as shown above.

The Royal Society of Chemistry apologises for these errors and any consequent inconvenience to authors and readers.

## Supplementary Material

